# Electrical/Mechanical Monitoring of Shape Memory Alloy Reinforcing Fibers Obtained by Pullout Tests in SMA/Cement Composite Materials

**DOI:** 10.3390/ma11020315

**Published:** 2018-02-22

**Authors:** Eui-Hyun Kim, Hyunbae Lee, Jae-Hwan Kim, Seung-Muk Bae, Heesu Hwang, Heesun Yang, Eunsoo Choi, Jin-Ha Hwang

**Affiliations:** 1 Department of Materials Science and Engineering, Hongik University, Seoul 04066, Korea; ineehg@hanmail.net (E.-H.K.); hyunbae90@naver.com (H.L.); jaehwan730@daum.net (J.-H.K.); hshwang9797@gmail.com (H.H.); hyang@hongik.ac.kr (H.Y.); 2 Center for Research Facilities, Kunsan National University, Kunsan 54150, Korea; moki1492@kunsan.ac.kr; 3 Department of Civil Engineering, Hongik University, Seoul 04066, Korea

**Keywords:** smart materials, impedance spectroscopy, fiber pullout resistance, shape memory materials, geometric modification

## Abstract

Self-healing is an essential property of smart concrete structures. In contrast to other structural metals, shape memory alloys (SMAs) offer two unique effects: shape memory effects, and superelastic effects. Composites composed of SMA wires and conventional cements can overcome the mechanical weaknesses associated with tensile fractures in conventional concretes. Under specialized environments, the material interface between the cementitious component and the SMA materials plays an important role in achieving the enhanced mechanical performance and robustness of the SMA/cement interface. This material interface is traditionally evaluated in terms of mechanical aspects, i.e., strain–stress characteristics. However, the current work attempts to simultaneously characterize the mechanical load-displacement relationships synchronized with impedance spectroscopy as a function of displacement. Frequency-dependent impedance spectroscopy is tested as an in situ monitoring tool for structural variations in smart composites composed of non-conducting cementitious materials and conducting metals. The artificial geometry change in the SMA wires is associated with an improved anchoring action that is compatible with the smallest variation in resistance compared with prismatic SMA wires embedded into a cement matrix. The significant increase in resistance is interpreted to be associated with the slip of the SMA fibers following the elastic deformation and the debonding of the SMA fiber/matrix.

## 1. Introduction

Shape memory alloys (SMAs) provide two useful forms depending on their polymorphism as a function of temperature and external stress: a shape memory form, and a superelastic form. The shape memory form is a prescribed shape from heating, and the superelastic form allows a phase transformation from austenite (the high-temperature phase) to martensite (the low-temperature phase) simply by increasing the external stress with no temperature change [1,2]. Among various shape memory alloys, Nitinol (known as a metal alloy of nickel and titanium) has excellent electrical and mechanical properties, a long fatigue life, and high corrosion resistance [3,4,5]. Shape memory alloys are of increasing academic/industrial interest due to their diverse benefits, such as self-healing functions and the ability to enhance the mechanical strength and robustness of cement-based materials [6,7].

Fiber-reinforced cement concretes (FRCCs) have been employed in a wide range of structural applications due to their enhanced tensile strength and ductile tensile performance. FRCCs are manufactured using a variety of fibers, such as steel fibers, carbon fibers, and polymer fibers [8,9,10]. The presence of these functional fibers influences crack-based phenomena, such as crack generation and propagation. The current work uses Ni/Ti-based shape memory alloy fibers, which have high tensile strength and bending strength, in contrast to conventional steel fibers. A variety of different geometries are proposed in this work to control and maximize the mechanical behaviors of SMA fibers, specifically to obtain higher tensile strength. The pre-stress, active confinement, seismic resistance, and self-repair properties of SMA fibers or wires have been tested previously [6,7,11].

Impedance spectroscopy was initially applied to monitor the hydration of cement-based materials [12,13,14,15]. Impedance spectroscopy offers unique features compared to direct current-based characterizations, including: (i) the simultaneous characterization of dielectric and electrical parameters, such as dielectric constants and conductivities; (ii) the systematic separation of electrical/dielectric origins among bulk-based, interface (or boundary)-based, or electrode-associated responses; and (iii) the visualization of electrical/dielectric homogeneities between the constituent elements [16]. The above functions can be modeled and understood in terms of the corresponding equivalent circuit models composed of resistors, capacitors, inductors, or generally constant phase elements [16].

For application to in situ monitoring of mechanical information as well as large-scale deformation, cracks and fractures, impedance spectroscopy can be manipulated for the self-sensing that is required before self-repairing (or self-healing) functions, which is crucial for preventive measures in emergency situations, and for increasing the structural durability and the survivability of earthquakes and typhoons [14,15,17]. The current work placed its main emphasis on the electrical self-sensing features that can be integrated with the self-healing function of the SMA materials using the shape memory effects through heating in smart concrete structures. This work also investigated the pullout resistances of artificially deformed SMA fibers embedded in cement mortar systems based on mechanical and electrical characteristics. The artificial modification of SMA fiber wires is expected to increase the bond resistance of fibers embedded into cement-based systems. The mechanical load-displacement relationship was synchronized with frequency-dependent impedance spectroscopy. In contrast to previous studies, SMA wires were employed in the present work as an electrode against the electrodes embedded in the cement-based matrices. The mechanical contact between the SMA wires and the cement-based materials affects the electrical conduction that forms between the two metal electrodes.

## 2. Experimental

**Figure 1:**
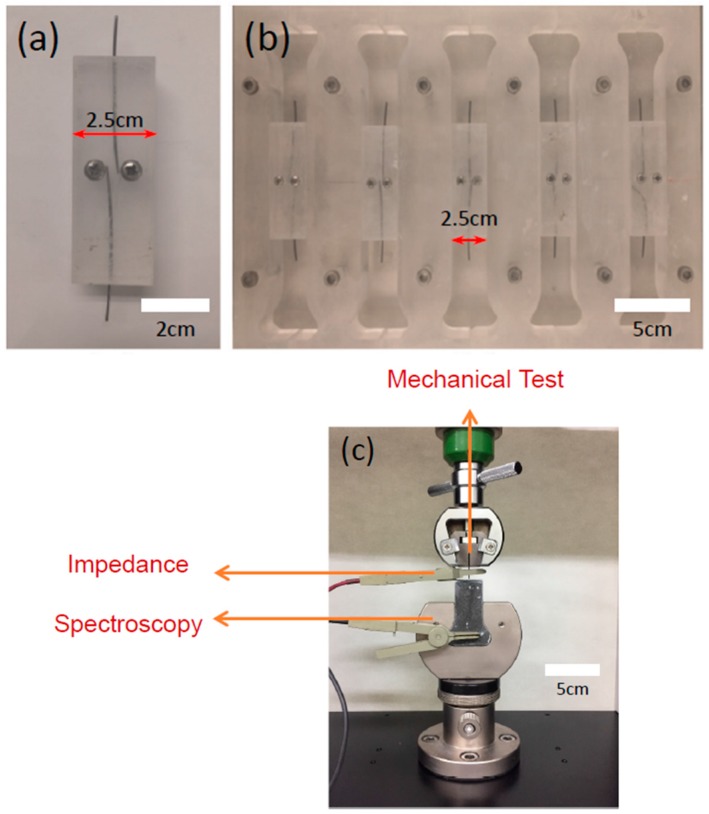
Molds for pullout specimens: (**a**) device for holding Ni/Ti shape memory alloy (SMA) fibers and (**b**) mold employed for pullout specimens inserted with the Ni/Ti SMA fibers. (**c**) Image showing the measurement environment allowing for simultaneous mechanical and electrical characterization.

**Figure 2:**
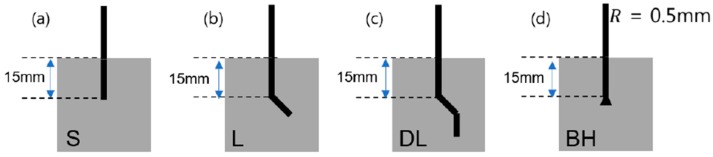
Shapes of Ni/Ti SMA wires employed in the pullout resistance characterization: (**a**) Straight SMA fibers (S-type); (**b**) L-shaped SMA fibers (L-type); (**c**) double-L shaped SMA fibers (DL-type); and (**d**) bulged-headed SMA fibers (BH-type).

Ni/Ti-based shape memory alloys were subjected to simultaneous mechanical and electrical characterizations using artificially-designed electrodes through modifications of the well-known mechanical apparatus for fiber pullout characterizations. The composition of the shape memory alloys employed in this work was Ni_50.4_Ti_49.6_, and the inserted SMA wires were cut into 30-mm wires with a diameter of approximately 1 mm. As shown in Figure 1, the SMA wires were partially embedded into the cement matrix at a depth of 15 mm, and the molds for producing the cement–fiber composite systems included an additional electrode for electrical characterization. In contrast to previous mechanical tests, the SMA fibers were employed as the top electrode, as well as the embedded electrode, with a depth of approximately 2.7 cm (the fibers were composed of steel foils with dimensions of 2 cm × 3 cm, and a thickness of 0.5 mm) during the load-displacement measurements. Straight SMA fibers were chosen as the reference materials (denoted by S-type SMA fibers). As illustrated in Figure 2, the fabricated geometries were L-shaped, double L-shaped, and bulged-headed SMA fibers which were denoted as L-type, DL-type, and BH-type SMA fibers, respectively. The SMA fibers functioned as one electrode for the impedance measurements.

For the simultaneous electrical and mechanical characterizations, the cement pastes were fabricated using ordinary Portland cement, fly ash, and silica fume, with the aim of mimicking practical concretes, despite the absence of sand and aggregates. The relative proportions of cement, fly ash, and silica fume were 74.8 wt %, 18.7 wt %, and 6.5 wt %, respectively. The water cement ratio (w/c) was controlled to a value of 0.37. The pastes were poured into the molds along with the SMA fibers and stainless steel electrodes according to the information given above. The test specimens were stored in a laboratory at room temperature and cured in water for 14 days.

Direct tensile tests were performed at ambient temperatures using a custom-made universal test machine under displacement control with a velocity of 2 mm/min until complete fracture at data acquisition rates of 4 Hz, i.e., four data points per second. Impedance spectra were acquired using a low-frequency impedance analyzer (HP 4192A, Hewlett-Packard, Palo Alto, CA, USA) with an oscillating voltage of 0.1 V between 1 MHz and 10 Hz, with 20 points per decade. The combined specimen and measurement apparatus is shown in Figure 1. The apparatus allowed both mechanical and electrical measurements without mutual interruptions. The electrical measurements were obtained using the two-point electrode configurations shown in Figure 1, where the top SMA wires functioned as an external electrode, and the bottom electrode was embedded into the cement mortars, which were fixed as a permanent electrode.

## 3. Results & Discussion

### 3.1. Mechanical Characterization of the NiTi SMA Fibers Embedded into the Cement Mortar

**Figure 3:**
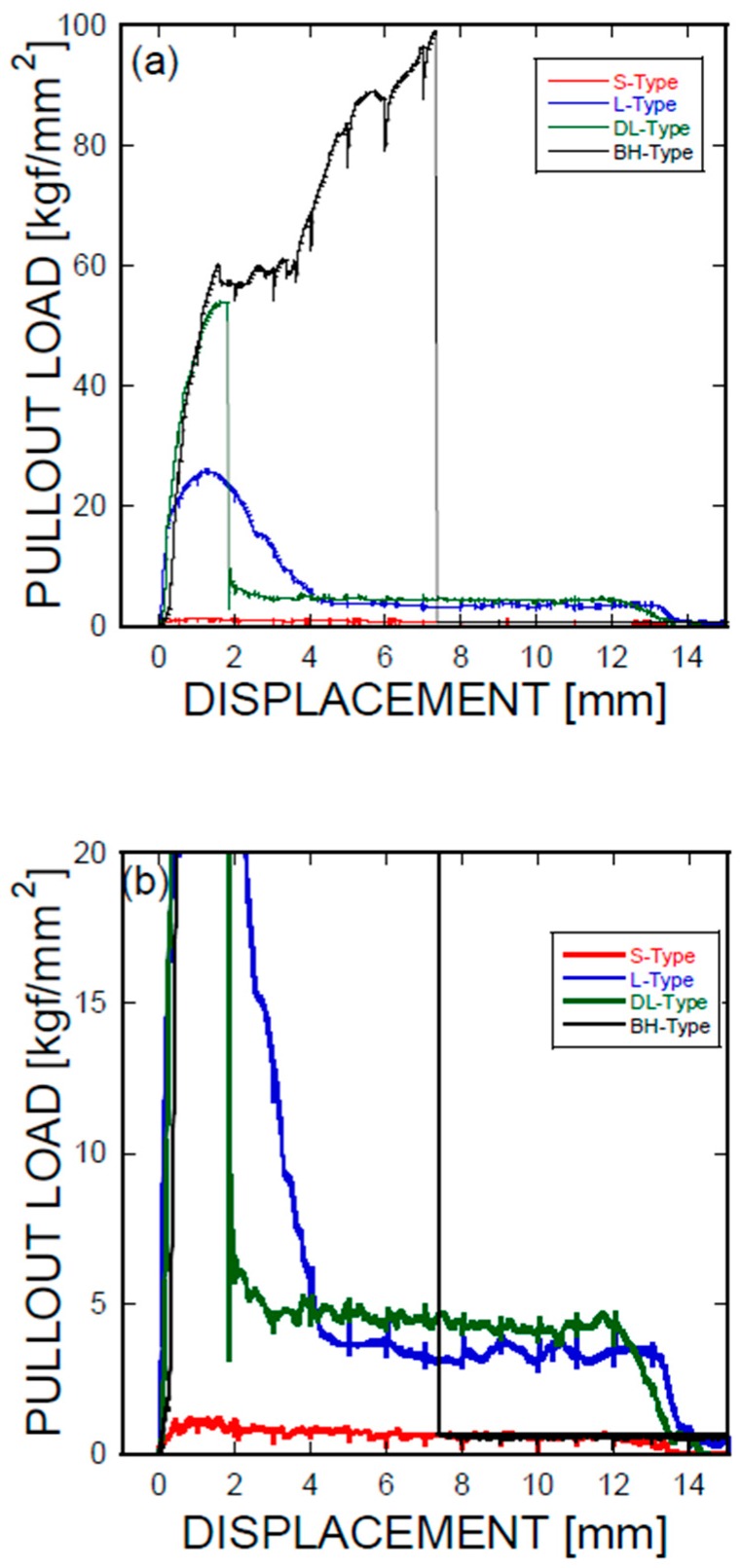
(**a**) Pullout load-displacement curves of Ni/Ti SMA fibers embedded in cementitious materials: S-type, L-type, DL-type, and BH-type; (**b**) an enlargement of Figure 3a acquired at the lower mechanical load.

Figure 3 shows the mechanical force-displacement behaviors associated with the pullout behaviors of the Ni/Ti SMA wires embedded into the cement-based matrix, in terms of the geometric shapes applied to the ends of the SMA fibers. An as-prepared S-type SMA fiber (Figure 2a and Figure 3) with no additional modifications at the ends of the SMA wires was used as a reference. The mechanical pullout force-displacement curves of the smooth, L-shaped, DL-shaped, and BH-shaped fibers are shown in Figure 3a,b, where Figure 3b is an enlargement of Figure 3a, and the lower and lowest mechanical resistances to the pullout of the SMA wires were identified. The detailed information is summarized in Table 1. According to Figure 3 and Table 1, the debonding started at short displacements, e.g., ranging from 0.68 mm to 1.56 mm. The DL and BH-shaped SMA fibers showed much higher displacements (with a factor of approximately two) than the straight SMA fiber, whose displacement was 0.68 mm.

**Figure 4:**
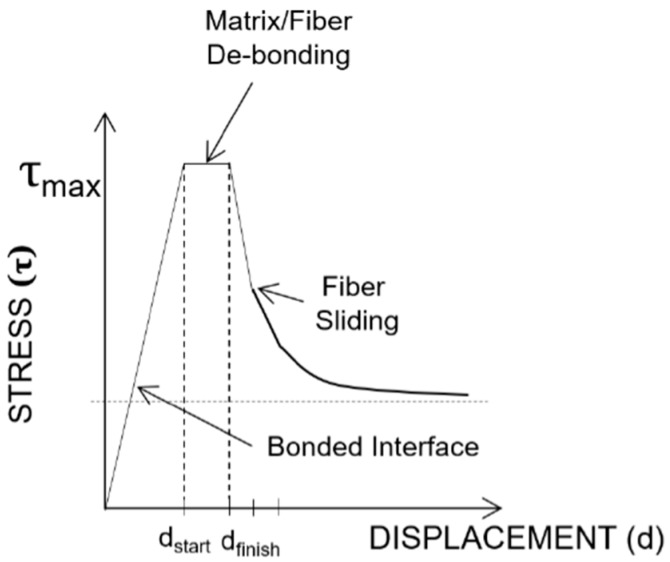
Schematic description showing the relationship between the applied stress and displacements in the pullout process of Ni/Ti SMA wires embedded into the cement matrix.

With the help of the schematic modified description of fiber pullout shown in Figure 4, which is based on the illustration proposed by Zhan and Meschke [18], the lowest pullout resistance in terms of mechanical performance was observed for the smooth fibers. The highest force (or stress) was 1.105, indicating no apparent pullout resistance due to the shape memory alloys. The largest pullout resistance was measured from the BH-shaped SMA wires, with a maximum load of 60.375 or 98.217 kg of force; a dual plateau was formed during the pullout test. The DL-shaped and L-shaped SMA fibers exhibited less pullout resistance, with maximum loads of 53.869 and 25.810 kg of force, respectively, which was lower than that of the BH-shaped SMA fibers. The resistance of the DL-shaped SMA fibers was greater than that of the L-shaped SMA fibers. As shown in Figure 3, the results for the straight SMA fibers were interpreted as slip that occurred immediately after debonding between the SMA fiber and the cement-based matrix. It is assumed that the mechanical fracture in the cement paste adjacent to the SMA fibers is considered to be minimal according to the comparison with the case involving the smooth SMA fiber. This issue should be addressed in the following investigation. However, the other three geometric modifications induced significant anchoring actions, especially in the BH-shaped SMAs (see Figure 3a,b). However, the mechanical behaviors can be classified depending on the presence of slip. The BH-shaped SMA fiber exhibited the largest pullout resistance, with a displacement of up to 7 mm, as demonstrated in Figure 3a,b. After that critical point, there was no detectable stress or applied forces, indicating that the SMA fibers were broken after the critical stress exceeding the fracture stress was applied to the SMA fibers. However, the results for the other two SMA fibers (L-shaped and DL-shaped) indicated that the frictional force played a role in the SMA pullout resistance, and that debonding was followed by frictional sliding.

### 3.2. Impedance-Based Characterization of the NiTi SMA Fibers Embedded in the Cement

**Figure 5:**
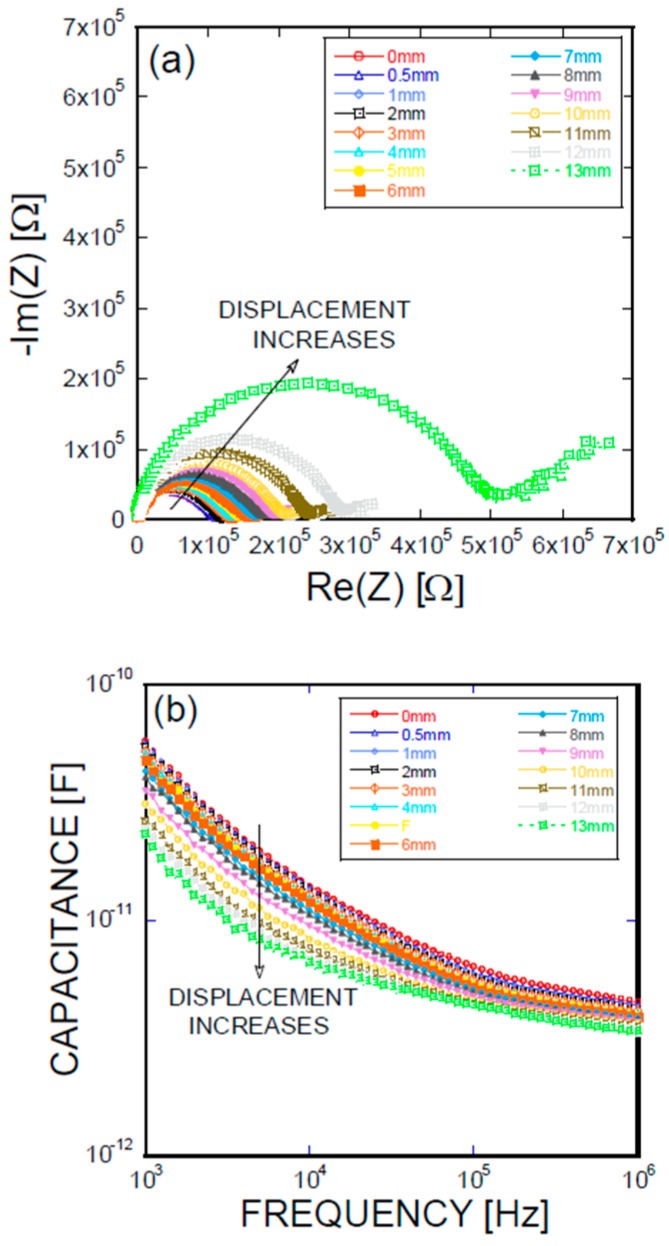
(**a**) Impedance spectra and capacitance Bode plots as a function of displacement obtained for an S-type Ni/Ti SMA fiber pullout; (**b**) mechanical load and electrical resistance extracted as a function of wire displacement obtained for an S-type Ni/Ti SMA fiber pullout.

**Figure 6:**
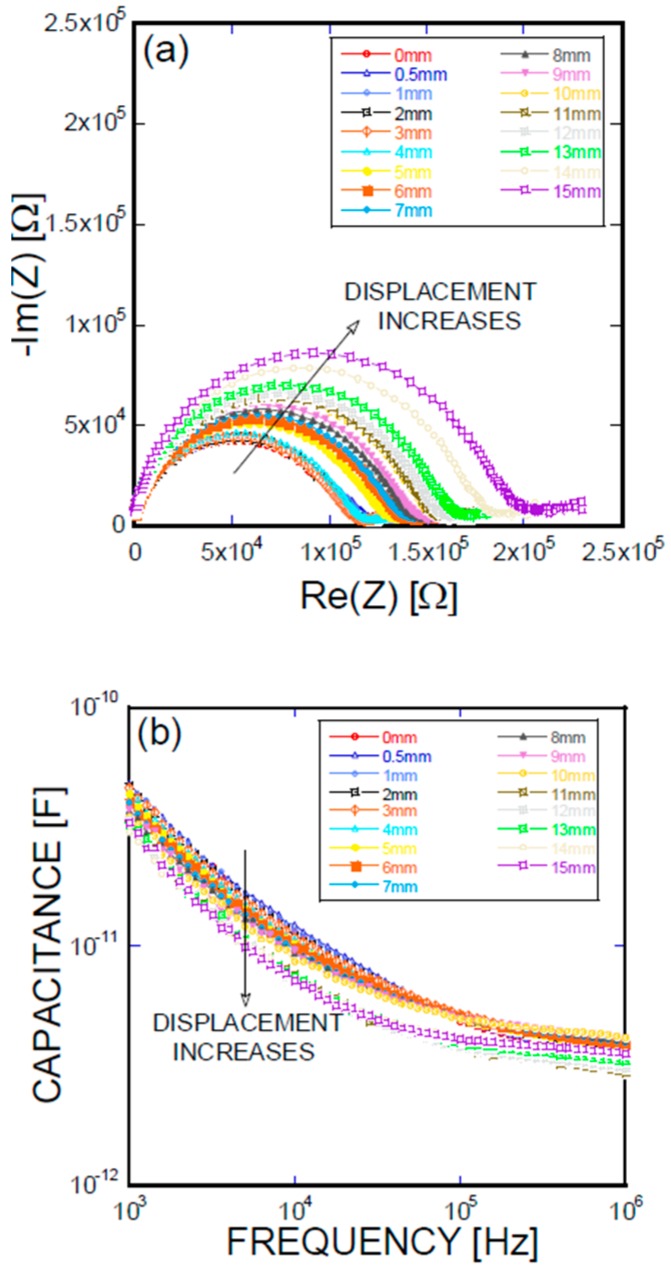
(**a**) Impedance spectra and capacitance Bode plots as a function of displacement obtained for an L-type Ni/Ti SMA fiber pullout; (**b**) mechanical load and electrical resistance extracted as a function of wire displacement obtained for an L-type Ni/Ti SMA fiber pullout.

**Figure 7:**
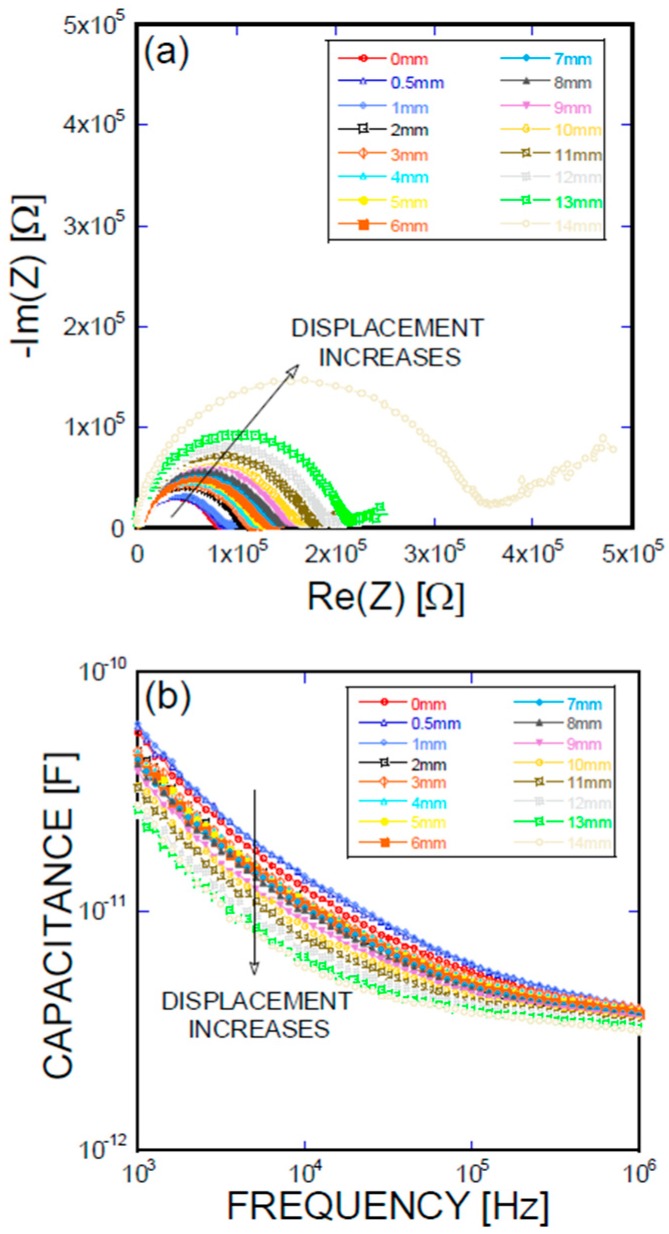
(**a**) Impedance spectra and capacitance Bode plots as a function of displacement obtained for a DL-type Ni/Ti SMA fiber pullout; (**b**) mechanical load and electrical resistance extracted as a function of wire displacement obtained for a DL-type Ni/Ti SMA fiber pullout.

**Figure 8:**
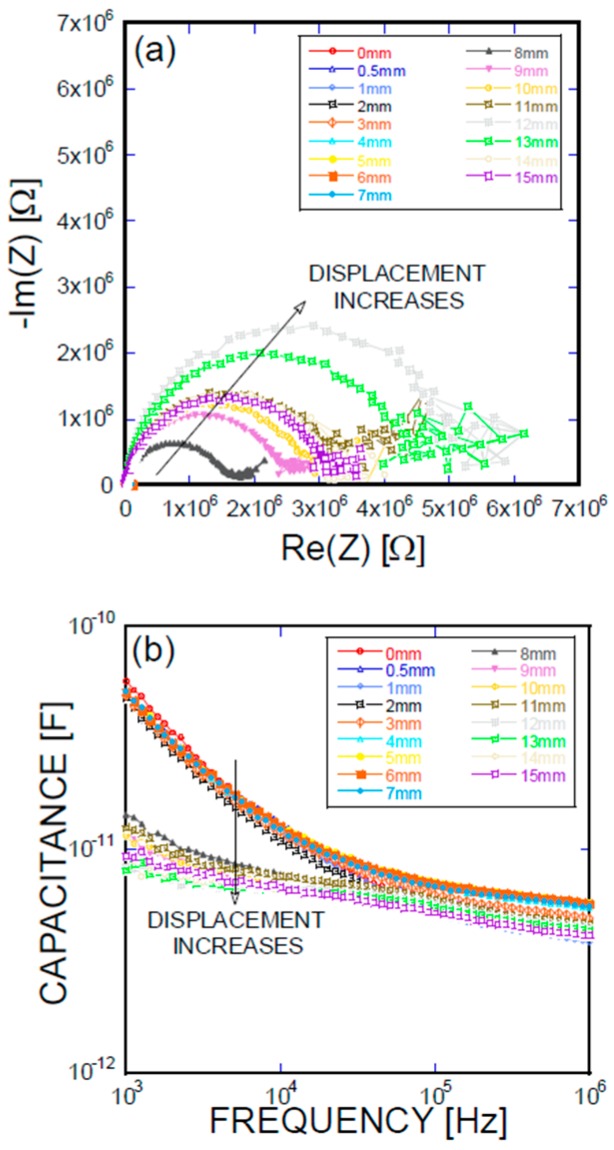
(**a**) Impedance spectra and capacitance Bode plots as a function of displacement obtained for a BH-type Ni/Ti SMA fiber pullout; (**b**) mechanical load and electrical resistance extracted as a function of wire displacement, obtained for a BH-type Ni/Ti SMA fiber pullout.

Figure 5, Figure 6, Figure 7 and Figure 8 present the empirical Nyquist impedance and capacitance Bode plots as a function of displacement in terms of Ni/Ti SMA geometries along with the reference SMA fiber. The figures correspond to the S-type fibers, L-type fibers, DL-type fibers, and BH-type fibers, respectively. The current impedance spectra can be approximately modeled as the serial connection of two parallel resistors and constant phase element circuits that are described as (R_hf_CPE_hf_) (R_lf_CPE_lf_), where R_hf_ and R_lf_ are high and low-frequency resistances, respectively, and CPE_hf_ and CPE_lf_ are high and low-frequency constant phase elements, respectively. A common point is the assumption that the low-frequency impedance arcs are attributable to the electrode polarization involving the charge transfer between metallic electrodes (i.e., SMA fibers, stainless steel plates, and cementitious materials). The complex capacitance can be converted from the measured impedance, which includes the real and imaginary impedance components, as given in the following equation [16]:

Re(C) = −Im(Z)/{(2πf(Re(Z)^2^ + Im(Z)^2^)} (1)

where f is the frequency of the applied AC(alternating current) voltage, Re(C) is the real component of capacitance, Re(Z) is the real component of complex impedance, and Im(Z) is the imaginary component of complex impedance. Figure 5a, Figure 6a, Figure 7a, and Figure 8a show the acquired impedance spectra, and Figure 5b, Figure 6b, Figure 7b, and Figure 8b provide the calculated capacitance that was obtained as a function of frequency and the geometric SMA shape. All four SMA geometries exhibited a decrease in capacitance with increasing displacement due to the decrease in charge-keeping capabilities. As demonstrated in the (real) capacitance Bode plots, the BH type in Figure 8b exhibits a discontinuity of the real capacitance, where the edge portions of the SMA ends are fractured, consequently leading to a facilitated pullout after the fracture of the SMA fiber. This excellent resistance is correlated with the lowest resistance between the SMA and the remaining electrode embedded in the cement-based matrices. The BH shape hinders the onset of fiber debonding; this behavior is in contrast to the S-type reference wire, which exhibits low resistance against the fiber, leading to higher electrical resistance.

### 3.3. Relationship between the Mechanical and Electrical Characterizations of the Ni/Ti SMA Fibers Embedded in the Cement

**Figure 9:**
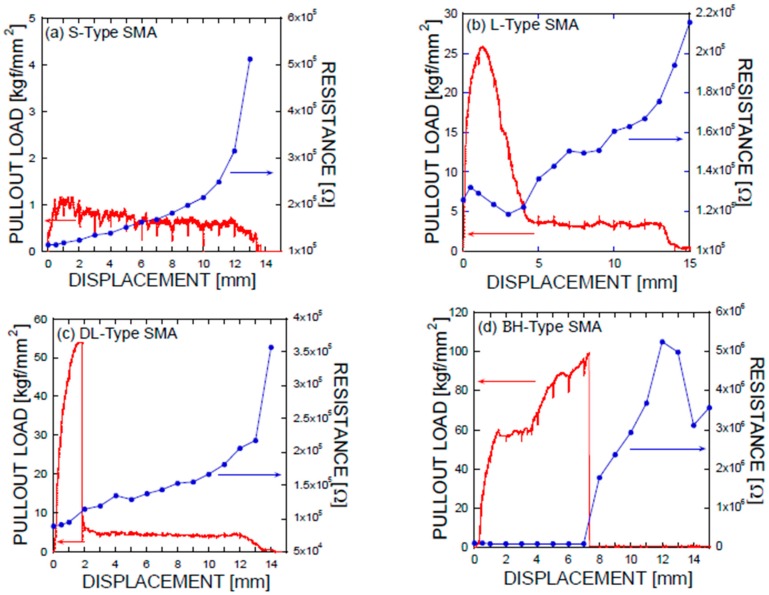
Simultaneous plot of electrical resistance and mechanical load obtained as a function of mechanical displacement obtained for (**a**) S-type; (**b**) L-type; (**c**) DL-type; and (**d**) BH-type Ni/Ti SMA fiber pullout tests.

An illustration of the simplified mechanical load-displacement phenomena provides a systematic understanding of the simultaneous complex plot, where the electrical resistance and loads are plotted as a function of displacement. The energy dissipation processes originate from the debonding, fiber-breaking failure, or fiber pullout [19]. The parameters affecting the bond stresses of a fiber are categorized into chemical adhesion, frictional adhesion, and anchoring action [20]. Figure 9 presents the electrical and mechanical resistance simultaneously as a function of mechanical displacement in terms of the geometrical shapes of the SMA fibers, or equivalently, anchorage geometries, along with a reference system in which the SMA fibers were embedded as prepared without any additional geometry change at the ends of the SMA fibers, i.e., the SMA fibers were straight and prismatic.

**Figure 10:**
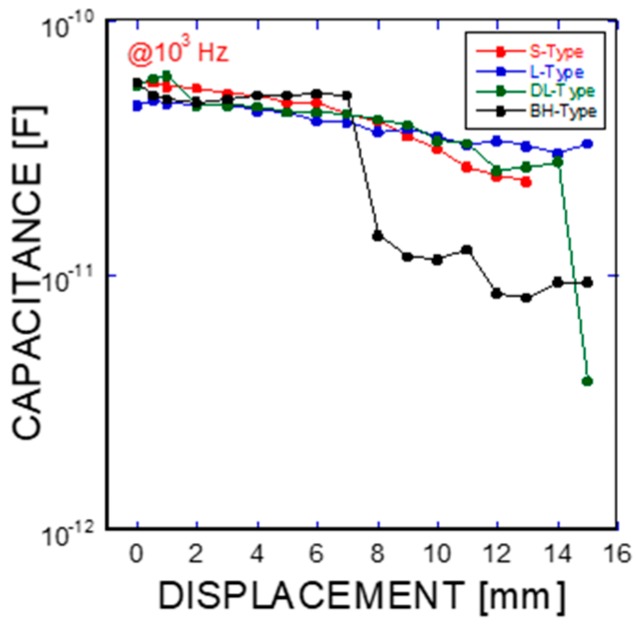
Capacitance change as a function of frequency in terms of the different SMA fiber geometries (S-type, L-type, DL-type, and BH-type Ni/Ti SMA fibers).

As shown in the pullout tests in Figure 3 and Figure 9, the prismatic shape of the SMA fibers did not induce any anchoring action. However, the artificially altered geometries produced anchoring effects, leading to a dramatic increase in the mechanical force applied (or equivalently, the stresses). Using the mechanical information on the applied load versus the displacement, integrations were performed among the proposed SMA geometries. Considering the cross-sectional area of the SMA fibers, the consumed energies are listed in terms of the relative values normalized by the total area covered by the mechanical load versus the displacement as shown in Figure 3a: 1 for the S-type fibers, 11.35 for the L-type fibers, 13.38 for the DL-type fibers, and 53.87 for the BH-type fibers. In other words, the mechanical resistance of the BH-type SMA fibers is approximately 54 times harder than the smooth SMA geometries from the perspective of consumed energy. The consumed mechanical energies for complete pullouts of the L-type and DL-type SMA fibers had intermediate values that were 10 times higher than those of the undeformed SMA fiber. The different pullout resistances are the result of the interplay of the tensile strength and stiffness of the SMA fibers in addition to the quality of the interfacial bonding formed between the smooth surface and the cement-based matrix [21,22]. The interfacial bond strength is shown in Figure 3a,b with the lowest mechanical load, in association with the debonding followed by the slide of the SMA fibers in the composite. However, the deformed SMA fibers are controlled by the tensile strength and the stiffness in terms of effective stress applied to the SMA fibers (see Figure 3 and Table 2). Additionally, the pullout processes of the SMA fibers embedded into cement-based matrixes have an influence on the charge capabilities, or equivalently, capacitances, as shown in Figure 10. The capacitances decrease with increasing displacement due to the concurrent decrease in the interfacial area formed between the cement-based matrix and the SMA fibers, which functions as an electrode. In particular, the drastic rupture of the SMA fibers invokes a discontinuous drop in the capacitance. However, resistance-based monitoring is more effective in tracking the displacement and interfacial robustness than capacitance-based monitoring. It should be noted that the significant increase in resistance-based monitoring is associated with the slip process that occurred in the SMA fiber pullout, as shown in Figure 9a–d.

The anchoring action of the SMA fibers is associated with the intimate contact between the SMA fiber and the surrounding cement matrix. The improved contact results in a gradual increase in the overall resistance. The most significant pullout resistance is associated with the bulge-headed shapes of SMA fibers, which are maintained for the longest time up to the fracture, while simultaneously maintaining the corresponding electrical resistance in the low-value state. The current impedance reflects the total contributions from the contact formed between the cement paste and the SMA fiber, and the mechanical damage of the cement paste. Depending on the SMA geometries, it is possible for the cement mortar to be damaged. However, the interaction between the SMA fiber and its adjacent cement paste is subjected to compressive stress. The mechanical strength of the cement paste is assumed to tolerate the external stimulus. If the mechanical damage occurs in the cement paste adjacent to the SMA fiber, then the energy dissipation can affect the pullout resistance and the subsequent electrical conductance. If there is any damage to the cement paste positioned near the SMA fiber, its effects are associated with an increase in electrical resistance. The contribution is reflected in the fiber/matrix debonding stages, following the initial elastic deformation in the mechanical load-displacement measurements. As shown in Figure 9, the whole SMA fibers did not show dramatic increases in electrical resistance, indicating that the effect of cement paste damage is minimal. The issue of the mechanical damage in the cement paste and the on-site monitoring confirmation should be investigated in further study.

## 4. Conclusions

This work successfully demonstrated the applicability of frequency-dependent impedance spectroscopy for pullout tests of functional fibers that are associated with the mechanical displacement-load relationship. Frequency-dependent impedance spectroscopy was applied to the mechanical monitoring of SMA pullout, with the aim of maximizing the pullout resistance of shape memory alloys. The artificially modified SMA wires exhibited mechanical resistance to the external pullout process, leading to enhanced electrical conductance: the highest electrical conductance was obtained in the double L-shaped (DL-type) and bulged-headed (BH-type) geometries, in contrast to that of the wire obtained from the straight wire (S-type). The optimized mechanical shape was consistent with the lowest electrical resistance, as shown in the BH-type SMA geometries. A delay in debonding between SMA wires and cement-based materials was successfully obtained for the bulged-head SMA fibers. This mechanical pullout resistance was characterized by the highest load in the load-displacement information and the highest electrical conduction, due to the intimate contact between the SMA wires and the cement materials. The significant increase in resistance is interpreted to be associated with the slip process occurring in the fiber pullout phenomena.

## Figures and Tables

**Table 1:** Summarized mechanical–electrical information obtained during the SMA pullout tests involving the geometric modifications in Ni/Ti SMA fibers: S-type, L-type, DL-type, and BH-type.

Displacement [mm]	S-Type		L-Type		DL-Type		BH-Type	
	Load [kgf]	Resistance [Ω]	Load [kgf]	Resistance [Ω]	Load [kgf]	Resistance [Ω]	Load [kgf]	Resistance [Ω]
0	0.000	1.14 × 10^5^	0.000	1.26 × 10^5^	0.000	8.85 × 10^4^	0.000	1.14 × 10^5^
0.5	0.900	1.14 × 10^5^	32.159	1.32 × 10^5^	32.159	9.06 × 10^4^	25.604	1.10 × 10^5^
1	1.105	1.18 × 10^5^	46.015	1.29 × 10^5^	46.015	9.49 × 10^4^	46.851	1.07 × 10^5^
2	0.977	1.24 × 10^5^	6.067	1.24 × 10^5^	6.067	1.14 × 10^5^	57.005	1.05 × 10^5^
3	0.810	1.34 × 10^5^	4.473	1.19 × 10^5^	4.473	1.19 × 10^5^	59.434	1.08 × 10^5^
4	0.835	1.39 × 10^5^	4.961	1.22 × 10^5^	4.961	1.34 × 10^5^	68.650	1.06 × 10^5^
5	0.746	1.51 × 10^5^	4.781	1.37 × 10^5^	4.781	1.29 × 10^5^	83.650	1.02 × 10^5^
6	0.630	1.62 × 10^5^	4.460	1.43 × 10^5^	4.460	1.38 × 10^5^	87.699	9.74 × 10^4^
7	0.630	1.69 × 10^5^	4.563	1.51 × 10^5^	4.563	1.43 × 10^5^	96.491	9.67 × 10^4^
8	0.514	1.83 × 10^5^	4.344	1.50 × 10^5^	4.344	1.53 × 10^5^	0.566	1.78 × 10^6^
9	0.643	1.99 × 10^5^	4.242	1.51 × 10^5^	4.242	1.55 × 10^5^	0.527	2.37 × 10^6^
10	0.476	2.15 × 10^5^	4.139	1.61 × 10^5^	4.139	1.67 × 10^5^	0.604	2.93 × 10^6^
11	0.540	2.48 × 10^5^	4.267	1.63 × 10^5^	4.267	1.82 × 10^5^	0.643	3.68 × 10^6^
12	0.656	3.15 × 10^5^	4.216	1.67 × 10^5^	4.216	2.05 × 10^5^	0.566	5.25 × 10^6^
13	0.386	5.12 × 10^5^	2.237	1.76 × 10^5^	2.237	2.18 × 10^5^	0.604	4.99 × 10^6^
14	0.000	-	0.540	1.94 × 10^5^	0.540	3.57 × 10^5^	0.566	3.12 × 10^6^
15	0.000	-	−0.167	2.16 × 10^5^	−0.167	-	0.591	3.57 × 10^6^

**Table 2:** Critical parameters extracted from the mechanical SMA pullout tests in terms of SMA geometric modifications. (d_start_: the mechanical displacement at the starting point of the debonding stage in the Ni/Ti SMA pullout test, d_finish_: the mechanical displacement at the finishing point of the debonding stage in the Ni/Ti SMA pullout test, τ_max_: the mechanical force (or stress) at the debonding stage).

Parameter	S-Type	L-Type	DL-Type	BH-Type
d_start_	0.68	1.19	1.56	1.54 or 5.26
d_finish_	1.59	1.35	1.82	3.63 or 6.00
τ_max_	1.157	25.810	53.869	60.375 or 88.217
